# Advances of MXenes; Perspectives on Biomedical Research

**DOI:** 10.3390/bios12070454

**Published:** 2022-06-25

**Authors:** Aneesh Koyappayil, Sachin Ganpat Chavan, Yun-Gil Roh, Min-Ho Lee

**Affiliations:** 1 School of Integrative Engineering, Chung-Ang University, 84 Heuseok-ro, Dongjak-Gu, Seoul 06974, Korea; aneesh@cau.ac.kr (A.K.); sachinchavan@cau.ac.kr (S.G.C.); 2 Department of Convergence in Health and Biomedicine, Chungbuk University, 1 Chungdae-ro, Seowon-gu, Cheongju 28644, Korea; shdbsrlf@chungbuk.ac.kr

**Keywords:** MXene, 2D materials, biomedical applications, transition metal carbides, transition metal nitrides

## Abstract

The last decade witnessed the emergence of a new family of 2D transition metal carbides and nitrides named MXenes, which quickly gained momentum due to their exceptional electrical, mechanical, optical, and tunable functionalities. These outstanding properties also rendered them attractive materials for biomedical and biosensing applications, including drug delivery systems, antimicrobial applications, tissue engineering, sensor probes, auxiliary agents for photothermal therapy and hyperthermia applications, etc. The hydrophilic nature of MXenes with rich surface functional groups is advantageous for biomedical applications over hydrophobic nanoparticles that may require complicated surface modifications. As an emerging 2D material with numerous phases and endless possible combinations with other 2D materials, 1D materials, nanoparticles, macromolecules, polymers, etc., MXenes opened a vast *terra incognita* for diverse biomedical applications. Recently, MXene research picked up the pace and resulted in a flood of literature reports with significant advancements in the biomedical field. In this context, this review will discuss the recent advancements, design principles, and working mechanisms of some interesting MXene-based biomedical applications. It also includes major progress, as well as key challenges of various types of MXenes and functional MXenes in conjugation with drug molecules, metallic nanoparticles, polymeric substrates, and other macromolecules. Finally, the future possibilities and challenges of this magnificent material are discussed in detail.

## 1. Introduction

**Figure 1:**
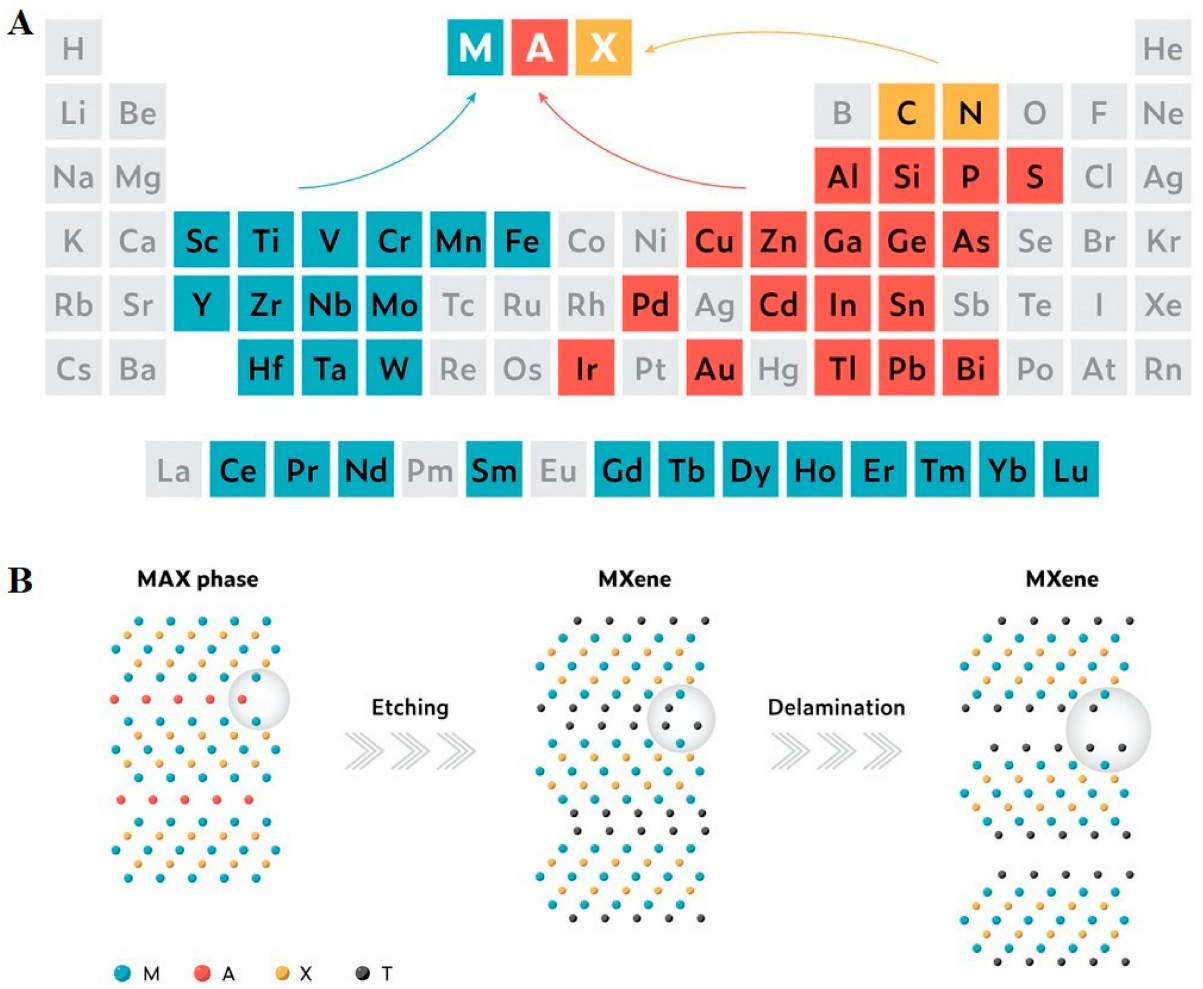
(**A**) The periodic table highlighting the ‘M’, ‘A’, and ‘X’ elements of known MAX phases. (**B**) Schematic illustration of the synthesis of MXenes from MAX phases. Reprinted with permission from Ref. [46]. Copyright © 2022 Wiley.

**Scheme 1:**
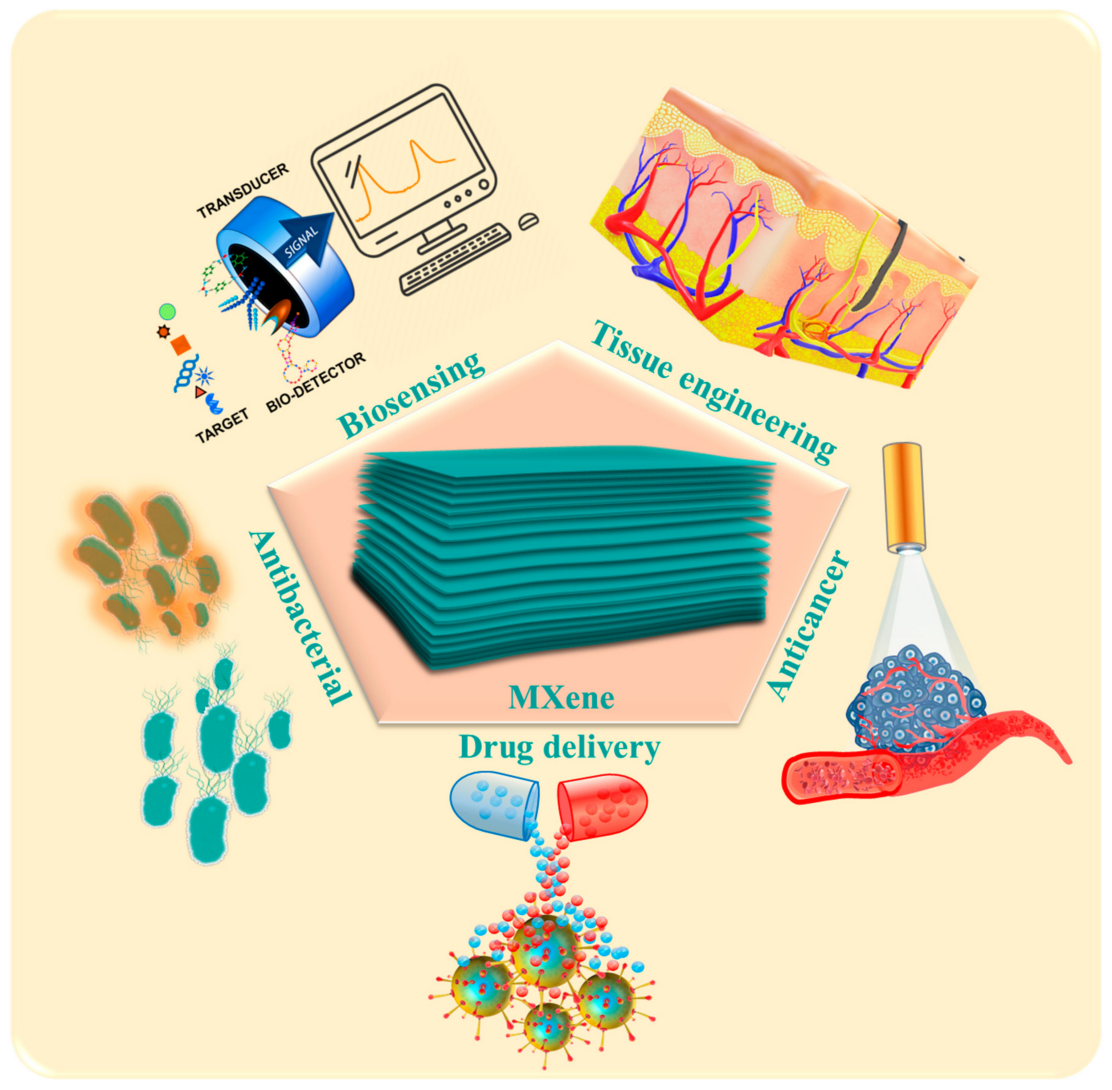
Biomedical applications of MXenes.

Research on 2D materials can be traced back to the pioneering work of Langmuir on elemental monolayers in the 1930s [1]. The long-forgotten research area underwent a reawakening with the discovery of graphene, the first two-dimensional atomic crystal, in 2004 [2,3], and its profound success thereafter. Since then, 2D materials such as hexagonal boron nitride, transition metal dichalcogenides, phosphorenes, etc. have been discovered and explored for promising applications [4]. MXenes (pronounced ‘maxenes’) emerged as an elegant member of the above category and soon proved to be versatile enough to revolutionize many aspects of human life by replacing some of the commonly used 2D materials to become the next disruptive technology. MXenes are synthesized from ‘MAX’ phases by the selective etching of ‘A’ layers. The MAX phases are conductive 2D layers of transition metal carbides/nitrides interconnected by the ‘A’ element with strong ionic, metallic, and covalent bonds [5]. As shown in Figure 1, a typical MXene 2D flake is formed by transition elements such as Sc, Ti, V, Cr, Mn, Fe, Y, Zr, Nb, Mo, Hf, Ta, and W interleaved by carbon or nitrogen with the general formula M_n+1_X_n_T_x_, where T_x_ represents surface functionalities such as F, Cl, O, and OH [6,7,8]. The history of MXenes begins in the year 2011 with the synthesis of 2D-layered Ti_3_C_2_T_x_ from the exfoliation of Ti_3_AlC_2_ MAX phase by Gogotsi‘s group [9]. The initial synthesis approach was conceptualized based on the weak Ti-Al metallic bond. This enabled the easy removal of Al atoms from the Ti_3_AlC_2_ MAX phase, such as AlF_3_, which was later removed by simple washing and resulted in a multilayered, accordion-like structure. This etching process was widely explored for the synthesis of different MXenes, and parameters such as etching time and HF concentration were optimized [10,11]. Owing to the high risk in handling and the corrosive nature of HF, several lower-risk alternative approaches have been conceptualized. Some of these approaches involved chemicals or combinations of chemicals such as NH_4_HF_2_ [12], HCl/FeF_3_ [13], HCl/LiF [14], HCl/NaF [15], HCl/KF [16], HCl/NH_4_F/KF [17], and HCl/NH_4_F [18], which can act as an in situ source of fluoride ions and improve the safety in operation to a large extent. Nowadays, fluorine-free synthesis approaches are gaining momentum as a new, safer gateway to MXene synthesis, and many innovative top-down synthesis routes, such as electrochemical etching [19,20], thermally assisted electrochemical approaches [21], hydrothermal treatments in NaOH [22] and KOH solutions [23], element replacement by reaction with Lewis acid molten salts [24], salt-templated approaches [25], etc., have been introduced. Moreover, bottom-up synthesis by chemical vapor deposition (CVD), plasma-enhanced pulsed laser deposition (PEPLD), and template methods [26,27] were also reported for the synthesis of MXenes. Because of their 2D planar structure, hydrophilicity, endless and flexible functionalization possibilities, strong absorption in the near-infrared (NIR) region, and exceptional properties, biomedical applications emerged as one of the most promising application fields of MXenes (Scheme 1). MXenes are found to be suitable candidates for applications including anticancer and drug delivery, antimicrobial, photothermal therapy, biosensors, and tissue engineering. However, even with intensive research efforts on MXene, the outstanding properties of these materials alone still cannot meet all the requirements of various biomedical applications. To endow new functions and to improve the performance, MXenes were functionalized, and their surfaces modified. Recently, functional modification of MXenes and the combination of MXenes with 3D [28], 2D [29], 1D [30], 0D [31], and polymer materials [32] with covalent and non-covalent modifications opened a new horizon for the functional requirements of biomedical applications. MXenes were modified with heteroatoms such as sulfur [33], phosphorous [34], and nitrogen [35] to produce functional MXenes. Apart from this, MXenes with enhanced properties were synthesized by doping with boron [36], platinum [37], niobium [38], silicon and germanium [39], vanadium [40], and alkali and alkaline earth metal cations [41]. As an ideal biomaterial for biomedical applications, MXenes, and their composites could be engineered with different physical, mechanical, or chemical properties [42], and must be compatible with the physiological environment with reliable mechanical strength, degradability, and the ability to overcome biological rejection [43]. Even though they have been less explored, several MXenes and their composites have proven to be biocompatible and non-toxic to living organisms [44], and MXenes such as niobium carbide have proven to be biodegradable in mice [45], thereby proving promising for in vivo applications.

In this review, we will focus on emphasizing the important biomedical applications of various MXenes, functional MXenes, and their composites, such as anticancer, drug delivery, antimicrobial, smart sensors, biosensors, and tissue engineering. Finally, the current challenges and prospects of MXenes, in this context, will be elaborated on in the conclusions section.

## 2. Drug Delivery Applications

**Figure 2:**
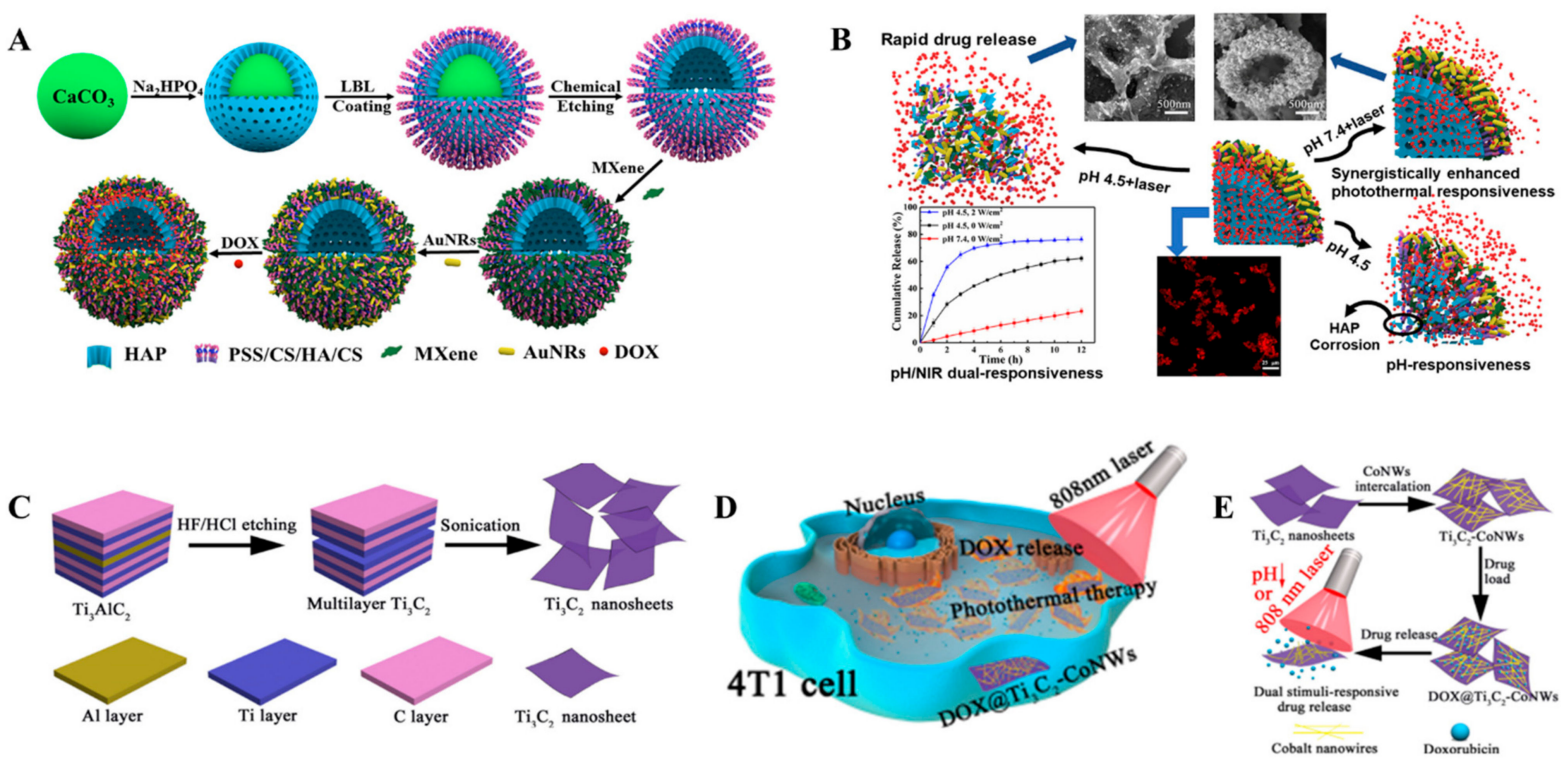
Schematic illustration of (**A**) Preparation of HAP/CS/HA/MXene/AuNRs microcapsules, and (**B**) Mechanism of pH/NIR-responsive drug release. Reprinted with permission from Ref. [54]. Copyright © 2021 Elsevier. (**C**) Schematic diagram for the synthesis of Ti_3_C_2_T_x_ nanosheets, (**D**) Dual-responsive drug release from Ti_3_C_2_T_x_-CoNWs system, and (**E**) Chemo-photothermal therapy of Ti_3_C_2_T_x_-CoNWs against cancer cells. Reprinted with permission from Ref. [49]. Copyright © 2020 Elsevier.

The unique 2D planar structure and physicochemical properties of MXenes make them favorable for precision drug delivery applications [47]. Ti_3_C_2_T_x_, a prominent member of the MXene family, is renowned for its drug delivery applications (Table 1) because of its ultrathin planar nanostructure, excellent photothermal conversion capability, excellent near-infrared (NIR) responsiveness, and the chemically tunable nature of the surface functionalities [48,49,50]. An ideal drug delivery system requires controllability and sufficient drug-loading capability so that the drug-carrying nanovehicles continuously stay in the required body part. However, Ti_3_C_2_T_x_-based nanovehicles lack sufficient controllability and suffer low drug-loading capability, so that, with blood circulation, the drug-carrying nanovehicles are continuously removed from the body site of application and cause inevitable damage to normal tissue [51]. Similar to other inorganic 2D materials, MXene-based nanoplatforms suffer stability in physiological conditions [52], which may affect the controllable release of the drug for cancer therapy. Therefore, fabricating a smart MXene-based nanoplatform for drug delivery remained a challenge. Controllability of a MXene-based drug carrier may be improved by adding magnetic nanomaterials so that the drug carrier could be controlled and confined to the target cells by the application of an external magnetic field. The drug, then, will be released by an endogenous or exogenous stimulation [53]. A few studies demonstrated the high drug-loading capability of MXene-metal oxide composites. Due to their successful therapy on cancerous cells, cobalt-based nanomaterials have attracted recent attraction. As a potent chemotherapeutic drug, doxorubicin (DOX) is usually taken as a model drug for drug delivery applications. An interesting dual-responsive DOX release was reported with the Ti_3_C_2_T_x_-CoNWs nanocarrier heterojunction [49] (Figure 2C–E). Here, the DOX release was triggered by the near-infrared (NIR) irradiation or acidic pH value (4–6). Similarly, Cao et al. [54] demonstrated a dual responsive (pH/NIR) drug delivery system consisting of layer by layer (LbL)-deposited hollow hydroxyapatite, chitosan/hyaluronic acid, AuNRs, and MXene. Here, the burst release of the drug in the initial stage of drug delivery was achieved using the HAP-deposited chitosan/hyaluronic acid, while the AuNRs and MXene on the surface of the LbL-deposited hybrid enhanced the photothermal conversion efficiency (Figure 2A,B). A similar strategy was adopted by Zhong et al. [55], and a MXene-hydrogel-based multiple-stimuli responsive drug-delivery system with photo- and magnetic-responsive properties was developed. In their experiment, the NIR and AMF-generated heat on the MNPs@MXene system triggered a shrinking process, and thus, drug release was made possible.

**Figure 3:**
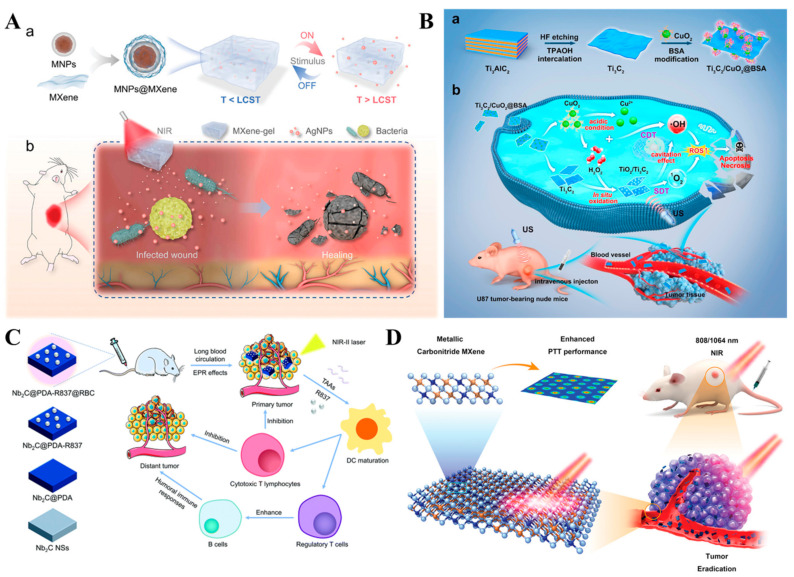
Schematic illustrations of (**A**) Functioning of stimuli-responsive MXene-hydrogel system. Drug release process of MXene-hydrogel (**a**), and deep chronic infected wound treated with MXene-hydrogel loaded with AgNPs (**b**). Reprinted with permission from Ref. [55]. Copyright © 2021 Wiley. (**B**) Synthesis of Ti_3_C_2_T_x_/CuO_2_@BSA nanosheets for the generation of in situ nanosensitizer for sonodynamic tumor nanotherapy. Reprinted with permission from Ref. [75]. Copyright © 2022 American Chemical Society. (**C**) Multifunctional nanoplatform based on Nb_2_C for the NIR-II-induced photothermal/immune therapy for primary as well as recurrent cancer. Reprinted with permission from Ref. [77]. Copyright © 2021 Royal Society of Chemistry. (**D**) Ti_3_CN-based NIR-I- and NIR-II-induced photonic hyperthermia against in vivo tumors. Reprinted with permission from Ref. [78]. Copyright © 2021 Wiley.

A similar kind of photo and magnetic dual-stimuli responsive MXene-hydrogel-based system for drug delivery and wound healing applications was reported by Zhong et al. [55] (Figure 3A). In the experiment, a hydrogel of poly (N-isopropyl acrylamide)-alginate and MXene-wrapped magnetic colloids were able to achieve a controlled drug delivery with reduced toxic side effects. The system was reportedly effective in the treatment of full-thickness cutaneous and subcutaneous wounds.

## 3. Anticancer Therapy

The disadvantages of conventional cancer therapy such as adverse/unwanted side effects, poor drug availability, requirement of high doses, indiscriminate targeting, and multidrug resistance [63,64] prompted intense research for effective cancer therapy and innovative nanosystems and drug formulations with less severe side effects [65,66] (Figure 3). Ti_3_C_2_T_x_-type MXenes were most extensively explored for anticancer applications [67], whereas MXenes such as Ti_2_C [68], Mo_2_C [69], Ta_4_C_3_ [70], Nb_2_C [71], TiCN [72], and V_2_C [73] were also reported for effective anticancer therapy. The outstanding photothermal performance of MXenes proved to be promising for anticancer photothermal therapy [67,74]. Additionally, it is possible to produce local hyperthermia by the application of a NIR irradiation, and with the combined effect of chemo-photothermal synergetic therapy, an effective and localized tumor eradication could be achieved (Table 2). Another way of achieving localized tumor eradication is by sonodynamic therapy. Chen et al. [75] reported a tumor microenvironment specific in situ generation of nanosonosensitizers on a Ti_3_C_2_T_x_/CuO_2_@BSA catalyst and achieved synergistic chemodynamic/sonodynamic tumor therapy. Cancer cells are known for an increased demand for iron, which is necessary for the early stages of metastasis, such as promoting cell proliferation and tumor progression, thereby making the iron metabolism an attractive therapeutic target for anticancer therapy. Jonathan et al. [76] reported an innovative iron-depletion-induced anti-tumor strategy based on the topoisomerase 2 inhibitor doxorubicin and iron chelator deferasirox (ExJade^®^) modified Ti_3_C_2_T_x_ MXene. The photoactivated Ti_3_C_2_T_x_-PVP@DOXjade reportedly downregulated the iron depletion-induced iron transferrin receptor and promoted apoptotic cell death. Wang et al. [61] reported a biodegradable organosilica-shell-coated Ti_2_N MXene as a biocompatible nanocarrier for tumor targeting. The tumor microenvironment activated pH, glutathione, and the photothermal-responsive drug release resulted in an effective dual-drug combination chemotherapy.

## 4. Antimicrobial Applications

**Figure 4:**
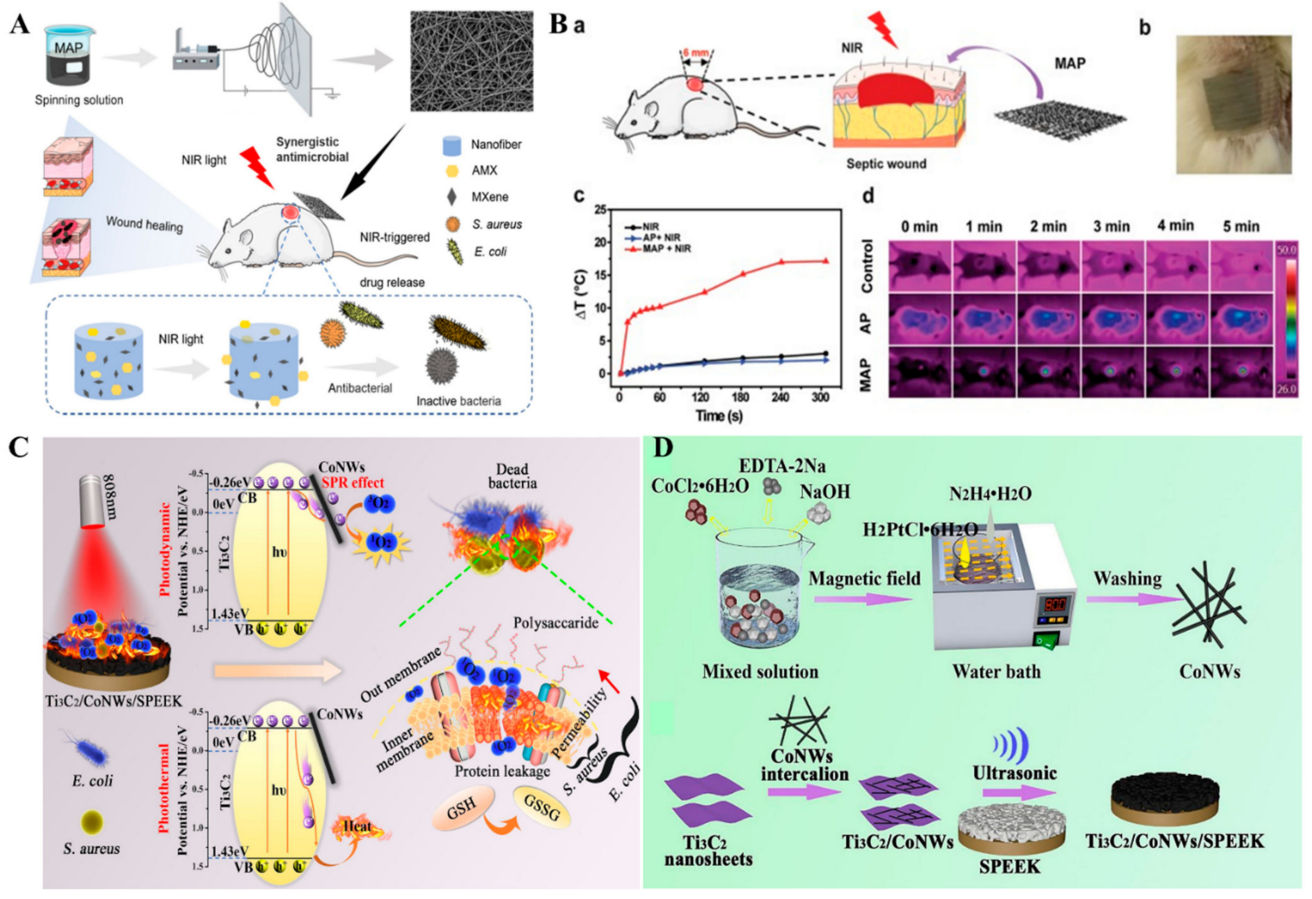
(**A**) Schematic illustration of the fabrication of MAP nanofibrous membrane for antimicrobial therapy. (**B**) A model of the Balb/c mice infected with *S. aureus* (**a**); An image of the antibacterial dressing using the MAP nanofibrous membrane (**b**); NIR-induced temperature increase in the *S. aureus*-infected mice wounds (**c**), and the corresponding thermal images (**d**). Reprinted with permission from Ref. [91]. Copyright © 2021 Elsevier. (**C**) Schematic diagram for the NIR-activated antimicrobial mechanism of Ti_3_C_2_T_x_/CoNWs/SPEEK. (**D**) Synthesis process for the coating of Ti_3_C_2_T_x_/CoNWs heterojunction on porous SPEEK. Reprinted with permission from Ref. [92]. Copyright © 2021 Elsevier.

Microbial growth is considered a serious health concern. Among various 2D materials, MXenes (particularly Ti_3_C_2_T_x_) have emerged as a promising candidate, showing antimicrobial activity even higher than graphene oxide [84]. MXenes have shown enhanced antimicrobial activity because of the enhanced cell membrane permeability, membrane rupture, DNA destruction because of the sharp edges, hydrophilicity, and hydrogen bonding with the cell membrane lipopolysaccharide molecules [85]. MXenes and their composites have shown excellent antibacterial properties against *Escherichia coli*, *Bacillus subtilis*, *Staphylococcus aureus*, *Pseudomonas aeruginosa*, and *Shigella* (Table 3). MXene functional groups are also reported to cause cell inactivation by preventing the intake of nutrients and thereby inhibiting the growth of bacteria [86]. The atomic structures of MXenes have been reported to have a crucial role in the antimicrobial properties of MXene [87]. Some MXenes such as Ti_3_C_2_T_x_ and TiVCT_x_ are reported to possess inherent antimicrobial properties. Additionally, the transfer of reactive electrons caused by the formation of a conductive bridge over the lipid bilayer from the bacterial cell to the external environment enables ultimate cell death [86]. Among the various other factors influencing the antibacterial efficiency of MXenes, environmental conditions and the structure of bacterial cell walls play a crucial role. Environmental conditions contribute to the aging of the membrane, and surface oxidation of Ti_3_C_2_T_x_ in the air results in the formation of nanocrystals of anatase TiO_2_ [88]. The TiO_2_ catalyzed free radical formation enhances the antibacterial property of Ti_3_C_2_T_x_ by stimulating oxidative stress on bacterial cell walls [88]. Since peptidoglycan thickness varies in gram-negative and gram-positive bacteria (peptidoglycan is thin in *E. coli* [89] and thick in *B. subtilis* [90]), a corresponding difference is observed in the resistance towards the MXene. Recently, the stoichiometry of the MXenes with the same chemical composition was also reported to exert considerable influence on its antibacterial activity [87]. In one study, Xua et al. [91] reported a multimodal antimicrobial platform based on MXene (Figure 4A,B). Amoxicillin, MXene, and polyvinyl alcohol were electrospun into a nanofibrous antibacterial membrane. In the study, the PVA matrix controlled the release of amoxicillin, whereas the MXene transformed the NIR laser into heat, leading to a local hyperthermia, which promoted the amoxicillin release. Ultimately, the synergistic effect of local hypothermia and amoxicillin caused the bacterial inactivation. The reported membrane not only functioned as a physical barrier to co-load the amoxicillin and MXene, but also exhibited a high antibacterial and accelerated wound-healing capacity. Deng et al. [92] reported an interesting NIR-activated MXene/cobalt nanowire 2D/1D heterojunction for antibacterial applications (Figure 4C,D). The heterojunction applied on an orthopedic implant achieved 90% antibacterial efficiency within 20 min because of the NIR-induced hyperthermia and ROS generation.

**Figure 5:**
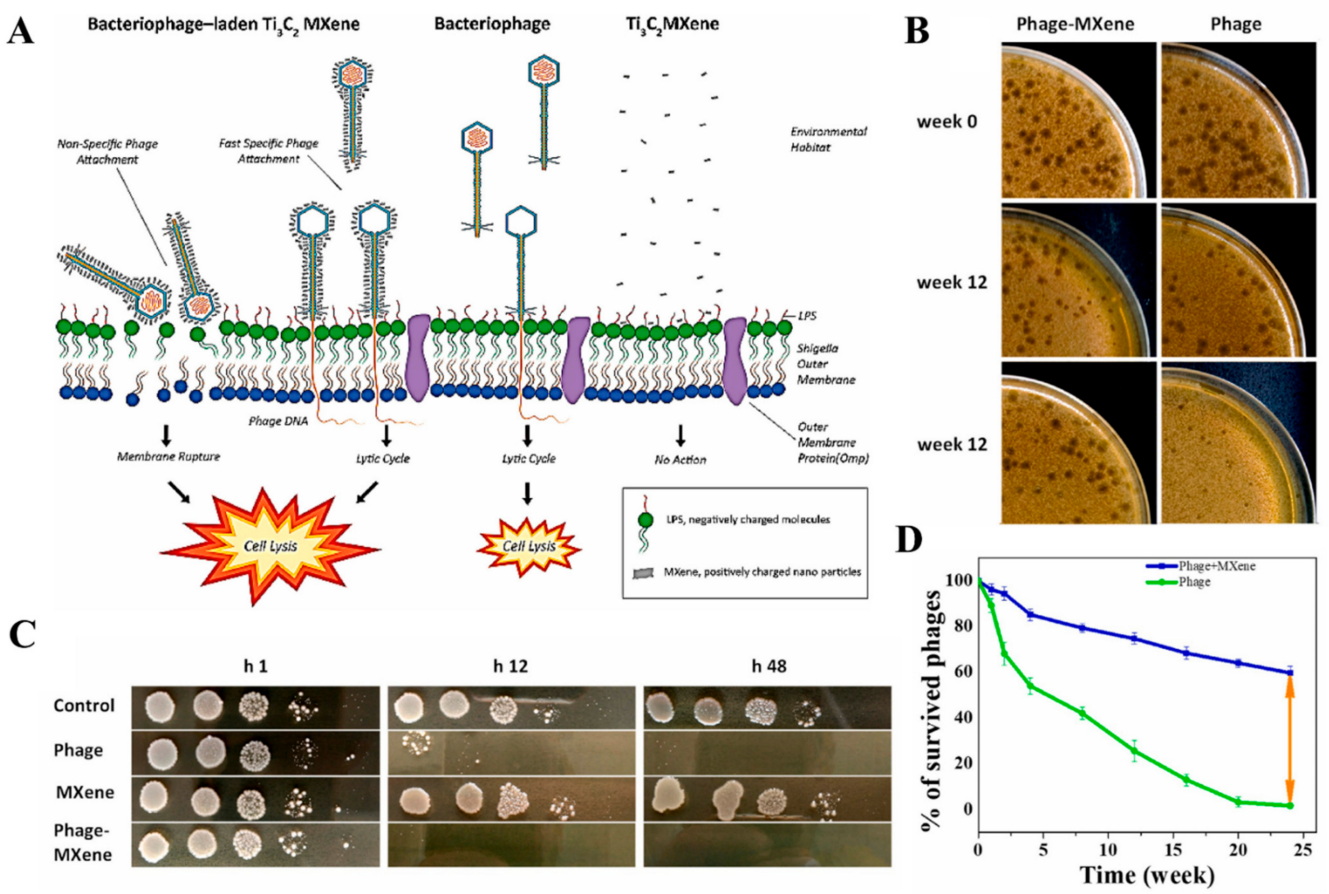
(**A**) Schematic illustration of the physical interaction of MXene-bacteriophage with bacteria leading to antimicrobial properties. (**B**) Pictures of bacteriophage spot assay indicating the improved stability of bacteriophage laden by MXene compared to the bacteriophage over different times. (**C**) Pictures of *Shigella* spot assay indicating the greater bacteria viability reduction in the presence of bacteriophage laden by Ti_3_C_2_T_x_ MXene compared to bacteriophage over different times. (**D**) The longevity of MXene-laden bacteriophage and free bacteriophage in water at 25 °C, over a period of 24 weeks incubation. Reprinted with permission from Ref. [100]. Copyright © 2022 Elsevier.

MXenes combined with metal oxides, polymers, nanoparticles, and bacteriophages also attracted significant research interest because of their enhanced antimicrobial properties. Pei et al. [100] reported an interesting scenario when the inherent antibacterial property of Ti_3_C_2_T_x_ was combined with the high specificity of a bacteriophage (Figure 5). In the study, the bacteria-targeting ability of bacteriophages combined with the physical interaction of MXene nanofragments and the bacterial cell membrane resulted in rupturing the cell wall, leading to microbial death. The results described that the Ti_3_C_2_T_x_ MXene significantly enhanced the bacteriophage adsorption rate and stability over long-standing cultivation in aquatic environments providing superior antibacterial efficacy against the bacterial target. MXene-laden bacteriophage reportedly reduced 99.99% of the artificial contamination in water samples.

Several MXene polymer composites have been reported to possess antimicrobial activity. Mahmoud et al. [108] reported PVDF-supported Ti_3_C_2_T_x_ for antimicrobial applications. The PVDF coating on the Ti_3_C_2_T_x_ improved the hydrophobicity in addition to alleviating the large pores in the membrane. The PVDF-MXene composite exerted 73% and 67% cell reduction on gram-negative *E. coli*, and gram-positive *B. subtilis* bacteria, respectively. Mayerberg et al. [95] developed biodegradable medical bandages with antimicrobial properties by electrospinning Ti_3_C_2_T_x_ MXene-chitosan. These electrospun nanofibers were stabilized by hydrogen bonding and electrostatic interactions between the positively charged chitosan functionalities and negatively charged MXene functional groups, and characterized by high porosity, permeability, absorptivity, and large surface area. The nanofibers exhibited 95% *E. coli* and 62% *S. aureus* cell reduction.

## 5. Biosensor and Smart Sensor Applications

Biosensors are receptor–transducer type devices that can convert a biological response to a readable output [109,110]. Recently, owing to the wide range of diagnosis applications for precise healthcare monitoring, the design and development of sensor materials have become center stage for the development of biosensors where advanced materials play a key role [111,112,113,114]. The advancement of technology has also driven smart and wearable devices for biomedical applications, and smart sensors on flexible electronics made great progress in medical applications [115,116,117]. Smart biosensors can respond to and record external stimuli such as electrical, chemical, optical, mechanical, and thermal, thereby enabling fitness tracking, real-time health monitoring, and disease forecasting [118,119]. Smart sensors are shifting the hospital-centered high-cost healthcare to homecare. The need for wearable smart sensors for biomedical applications is growing rapidly and is expected to reach a revenue of USD 97.8 billion by 2023 [120]. Nanomaterials have been explored extensively for smart sensor devices [121,122], and since its discovery, graphene has been utilized as a base material for a variety of smart devices [123,124]. Recently, MXenes with superior electrochemical properties emerged as a viable alternative to graphene in smart devices.

### 5.1. MXene-Based Smart Sensors

**Figure 6:**
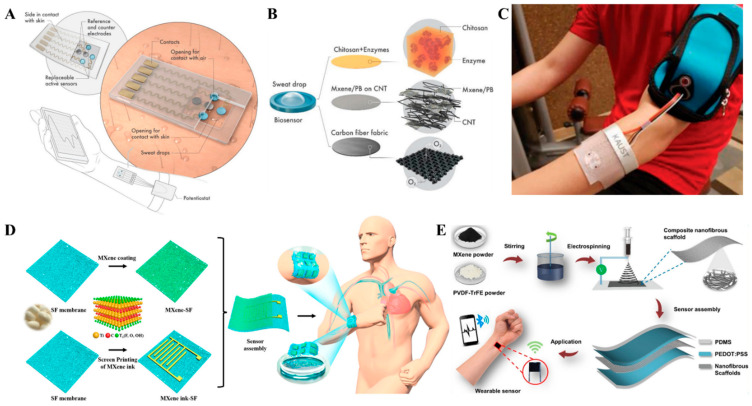
(**A**) Schematic illustration of the MXene/Prussian blue-based wearable perspiration analysis system. (**B**) Oxygen-rich enzyme electrode. (**C**) Digital photograph of the wearable sensor patch on the skin connected to a portable electrochemical analyzer. Reproduced with permission from Ref. [130]. Copyright © 2019 Wiley. (**D**) Schematic illustration of the fabrication of MXene/protein nanocomposite-based breathable and degradable pressure sensor. Reproduced with permission from Ref. [132]. Copyright © 2021 American Chemical Society. (**E**) Schematic illustration of the fabrication of MXene composite nanofibrous scaffold-based pressure sensor. Reproduced with permission from Ref. [133]. Copyright © 2020 American Chemical Society.

**Figure 7:**
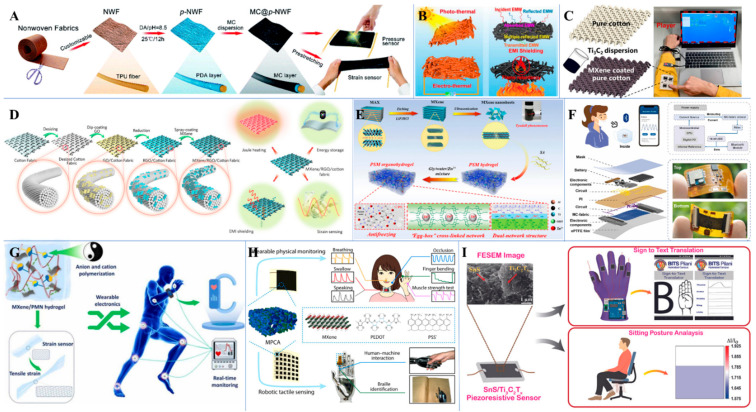
(**A**) Schematic diagram of the preparation of MXene@ polyurethane non-woven fabric for tunable wearable strain/pressure sensor. Reprinted with permission from Ref. [134]. Copyright © 2020 Royal Society of Chemistry. (**B**) Schematics of the MXene-coated non-woven fabric for wearable heater and EMI Shielding applications. Reprinted with permission from Ref. [135]. Copyright © 2022 Elsevier. (**C**) A MXene-coated cotton fabric for real-time pressure monitoring. Reprinted with permission from Ref. [136]. Copyright © 2020 American Chemical Society. (**D**) Schematic illustration of the fabrication of MXene/rGO cotton fabrics for strain sensing, EMI shielding, energy storage, and Joule heating applications. Reprinted with permission from Ref. [137]. Copyright © 2021 Elsevier. (**E**) Schematic illustration of the fabrication of PSM organohydrogels for wearable wireless human motion monitoring sensors. Reprinted with permission from Ref. [144]. Copyright © 2022 Elsevier. (**F**) Schematics of the overall design of MXene/MWCNT-based smart mask for respiration monitoring. Reprinted with permission from Ref. [131]. Copyright © 2022 Elsevier. (**G**) Schematic illustration of the fabrication process of stretchable and self-healing MXene/PMN hydrogel for wearable epidermal sensor applications. Reprinted with permission from Ref. [145]. Copyright © 2022 Elsevier. (**H**) Applications of 3D MXene/PEDOT: PSS-based pressure sensing devices. Reprinted with permission from Ref. [146]. Copyright © 2022 American Chemical Society. (**I**) Wearable electromechanical sensor based on SnS/Ti_3_C_2_T_x_ for sign-to-text translation and sitting posture analysis. Reprinted with permission from Ref. [138]. Copyright © 2022 American Chemical Society.

MXene-based smart devices attracted recent interest for potential applications in healthcare, fitness, EMG signal analysis, and human motion monitoring [125] (Figure 6 and Figure 7) because of their extreme thinness [126], transparency [127], and mechanical strength [128]. MXenes can be applied on cellulose by printing, spinning, plating, and dip coating or spray coating, which can later be integrated into garments for wearable applications [129]. Garments made from MXene-coated yarns are widely explored for fitness and healthcare monitoring (Table 4). An interesting MXene-based smart device was reported by Yongjiu et al. [130] for perspiration analysis. The MXene/Prussian blue composite electrode-based sensor with a unique modular and solid-liquid-air three-phase interface design enabled the detection of glucose and lactate from sweat (Figure 6A–C). The sensor was able to achieve a sensitivity of 11.4 µA/mMcm^2^ for lactate and 35.3 µA/mMcm^2^ for glucose using artificial sweat. Han et al. [131] designed an innovative smart mask with a built-in wireless data transmission system for real-time respiration monitoring based on Ti_3_C_2_T_x_/MWCNT (Figure 7F). The smart mask was able to accurately identify various respiration patterns with remarkable performance under deformation conditions and higher humidity conditions (265% response at a relative humidity of 90%). With the advancement of MXenes, research on MXene-based smart devices is also gaining momentum. However, MXene-based smart devices are in an infant stage when compared to other 2D materials like graphene, with only a handful of applications and devices.

### 5.2. Biomarker Detection

**Figure 8:**
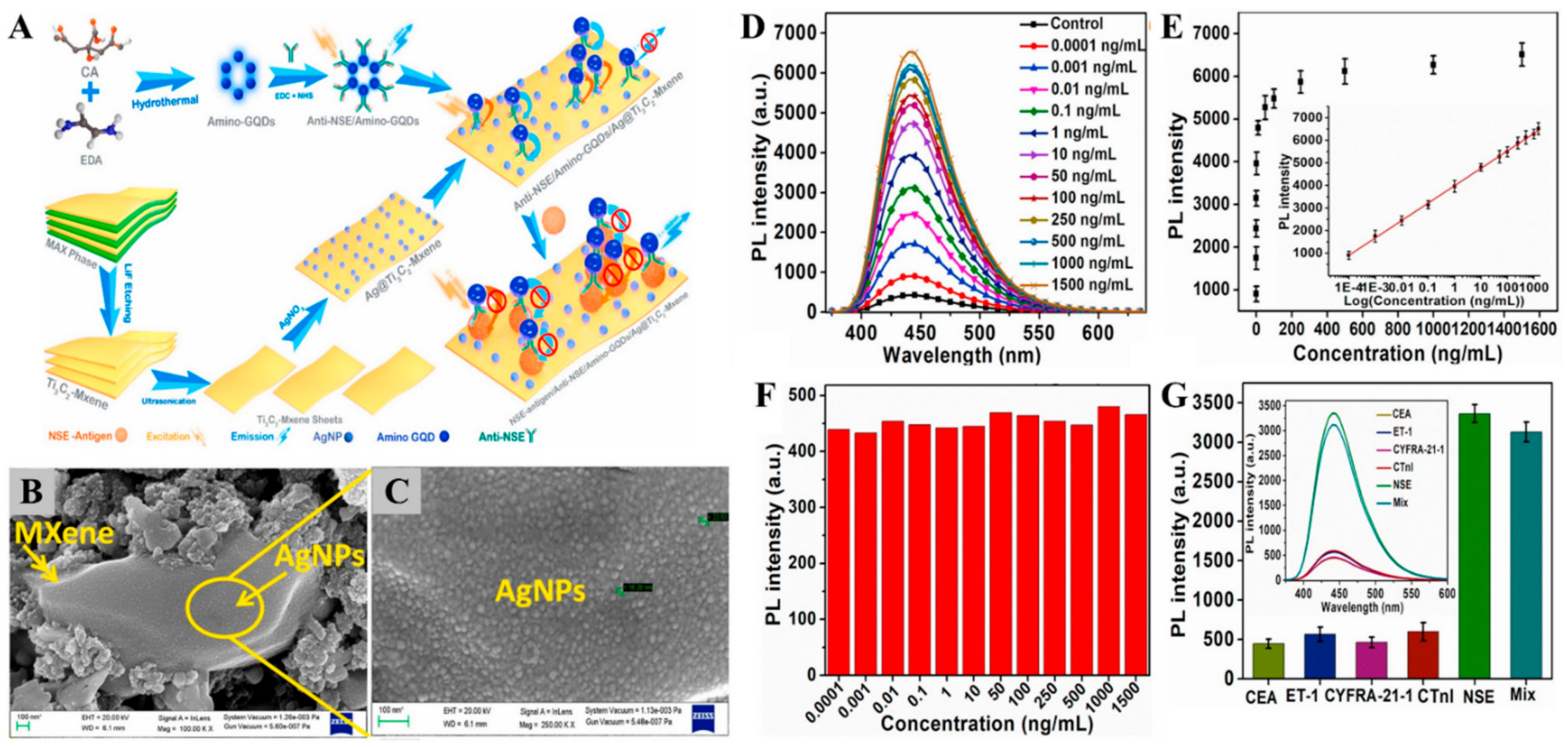
(**A**) Schematic illustration of the anti-NSE/amino-GQDs/Ag@Ti_3_C_2_T_x_ based fluorometric NSE detection. (**B**,**C**) FE-SEM images of Ag nanoparticles decorated Ti_3_C_2_T_x_ MXene at different magnifications. (**D**) Analytical performance of the anti-NSE/amino-GQDs/Ag@Ti_3_C_2_T_x_ based fluorescent biosensor toward NSE detection. (**E**) Graph showing the correlation between recovered fluorescence and NSE concentration, inset shows the corresponding calibration curve between recovered fluorescence and log NSE concentration in the range of 1 × 10^−4^–1.5 × 10^3^ ng/mL. (**F**) Selectivity of the proposed biosensor. (**G**) Control experiment for the amino-GQDs/Ag@Ti_3_C_2_T_x_ electrode. Reprinted with permission from Ref. [152]. Copyright © 2022 Elsevier.

Biomarker detection is regarded as an exquisite section of biosensors. A biomarker can be defined as any biomolecule or its products in the body that can be measured to predict the incidence of disease [147]. Biomarker detection is of great significance in disease screening, and early diagnosis and careful assessment of the validity of biomarkers are required concerning each stage of disease [148]. The most critical aspect in the development of biomarker-based biosensors is the ability to selectively detect the analytes of interest to diagnose the onset and progress of the disease. This makes medical intervention possible at an earlier stage for enhanced curative efficiency [149]. MXene 2D sensor platforms, the Ti_3_C_2_T_x_ MXene in particular, were extensively used for the detection of biomarkers up to attomolar concentrations (Table 5). An interesting MXene-based photothermal multi-signal readout sensor for the detection of the hepatitis biomarker human anti-ASGPR was reported by Chen et al. [150]. In the experiment, the Ti_3_C_2_T_x_@CuNCs induced visual color change from moderate blue to deep blue in the oxidized-reduced methylene blue (MB-MBH_2_) colorimetric system due to CuNCs catalyzed redox process of methylene blue. The Ti_3_C_2_T_x_@CuNCs/MB complex also presented a brilliant photo-to-thermal conversion ability under NIR laser radiation with an enhanced photothermal effect, thereby significantly amplifying the temperature in temperature-responsive soft electronic devices (SED). Additionally, since the conductivity variation in SED is influenced by the increased temperature, a provision for using a single digital multimeter (DMM) to convert the sensor response into a measurable electrical signal was obtained. MXenes have also been widely explored for anchoring antibodies and entrapping biomolecules owing to the high surface of the 2D nanolayer architecture [51]. The electrocatalytic properties of MXenes are altered by the active functional groups of immobilized molecules and result in the electrochemical signal. The rich active functional groups also support the biological receptors by covalent immobilization and contribute to enhanced biosensor performance. A similar strategy was adopted by Kumar et al. [151] for carcinoembryonic antigen (CEA) detection. In the experiment, Ti_3_C_2_T_x_ nanosheets functionalized with aminosilane, which were covalently bound to the bio-receptor anti-CEA for the label-free, sensitive detection of CEA. The sensor was able to achieve a remarkable linear range (1.0 × 10^−4^–2000 ng/mL) and detection limit (0.000018 ng/mL). Gopinath et al. [152] reported a MXene-based novel platform for the fluorimetric detection of biomarker neuron-specific enolase (Figure 8). In the study, the working principle relied upon the fluorescent quenching of an anti-NSE/amino-GQDs/Ag@Ti_3_C_2_T_x_-based fluorescent sensor in the presence of NSE. The sensor performance was remarkable, with a broad linear range (0.0001–1500 ng/mL), a better limit of detection (0.05 pg/mL), and a faster response time of 12 min. Furthermore, the sensor reported a fluorescence recovery of ~98% from real serum samples.

**Figure 9:**
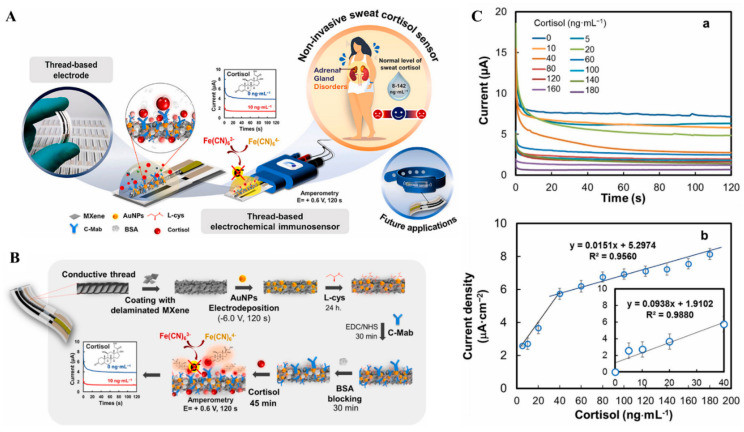
Schematic illustration of (**A**) MXene–based non-invasive sweat–cortisol sensor and (**B**) Thread–based electrochemical immunosensor fabrication for cortisol detection. (**C**) Amperometric responses for cortisol in the concentration range of 5–180 ng/mL at the applied potential +0.6 V (vs. Ag/AgCl) for 120 s (**a**). Calibration plot of the cortisol sensor (**b**). Reproduced with permission from Ref. [170]. Copyright © 2022 Elsevier.

In one study, a non-invasive electrochemical immunosensor for the detection of sweat cortisol, which is a potent biomarker for identifying adrenal gland disorders, was illustrated by Rodthongkum et al. [170]. In the study, thread-based working electrodes were fabricated with L-Cys/AuNPs/MXene (Figure 9). The AuNPs increased the sensitivity of the detection system by increasing the specific surface area, whereas MXene served to anchor monoclonal anti-cortisol antibodies. The antigen–antibody binding interaction and the decrease in current as a result of the blocking of the electron transfer process by cortisol resulted in an amperometric sensor for cortisol in the concentration range of 5–180 ng/mL.

### 5.3. Enzymatic Sensors

**Figure 10:**
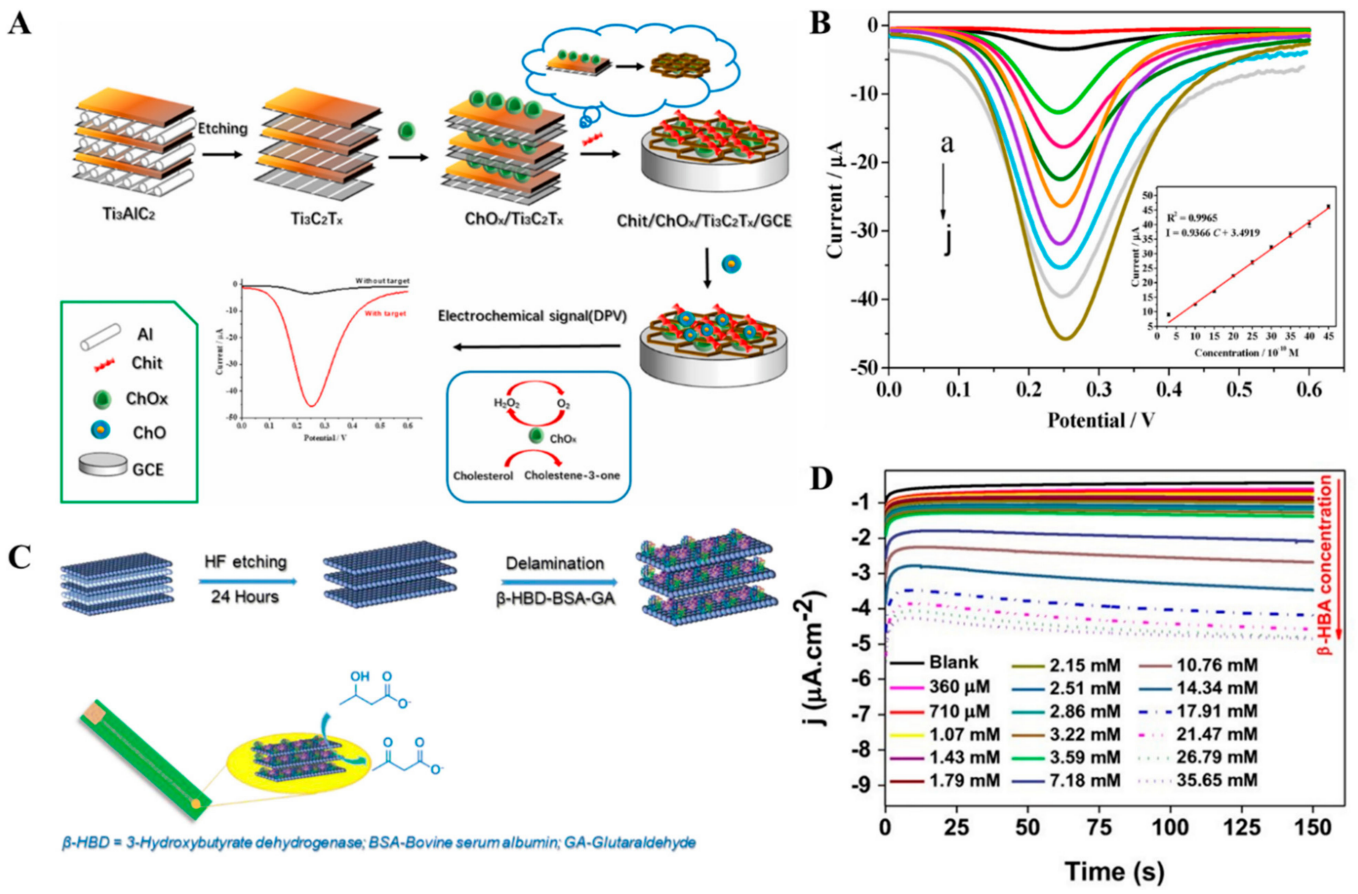
(**A**) Schematic illustration of the fabrication of Chit/ChO_x_/Ti_3_C_2_T_x_/GCE for the voltametric determination of cholesterol. (**B**) DPVs at the Chit/ChO_x_/Ti_3_C_2_T_x_/GCE in different cholesterol concentrations in the presence of 1 mM Fe(CN)_6_^3^^−^^/4^^−^ containing 0.1 M KCl. Inset is the corresponding calibration plot for cholesterol. Reprinted with permission from Ref. [153]. Copyright © 2021 Elsevier. (**C**) Schematic representation of the fabrication of MXene decorated β−hydroxybutyrate dehydrogenase. (**D**) Amperometric i−t curve at the Au-PCB/Ru/MXene−β−HBD−NAD^+^−GA−BSA electrode for the determination of β−HBA. Reprinted with permission from Ref. [172]. Copyright © 2020 Springer.

Two-dimensional multilayered MXene nanolayers with high surface area can provide a protective microenvironment for entrapping enzymes where they can maintain activity and stability [51,172]. The entrapped enzymes alter the electrocatalytic properties of the MXenes and provide a linear response for sensor applications. More importantly, MXene layers with concentrating effects on substrates improve the accessibility of immobilized enzymes with the substrates with an increased effective collision and enhanced sensor response. Moreover, MXenes are regarded as suitable candidates for biosensor applications because of their remarkable electronic, optical, and tunable chemical functionalities. MXenes with sufficient biocompatibility, metallic conductivity, and hydrophilic surface make them suitable for entrapping enzymes, and several enzymatic sensors are fabricated by entrapping enzymes such as β-hydroxybutyrate dehydrogenase [172], glucose oxidase [173], lactate oxidase [130], cholesterol oxidase [153], horseradish peroxidase [174], acetylcholinesterase [175], tyrosinase [176], and xanthine oxidase [177]. In one study, a voltametric cholesterol sensor was realized by the fabrication of Chit/ChOx/Ti_3_C_2_T_x_ through a continuous self-assembled process (Figure 10A,B). In the study, the chitosan and MXene helped to immobilize the enzyme while simultaneously improving the electronic conductivity, and thereby, the electron transfer rate [153]. Fan et al. [178] reported a covalent immobilization strategy for lipase onto Ti_3_C_2_T_x_ MXene. The resulting immobilized lipase exhibited excellent thermal and pH stability, and reusability.

## 6. Tissue Engineering

**Figure 11:**
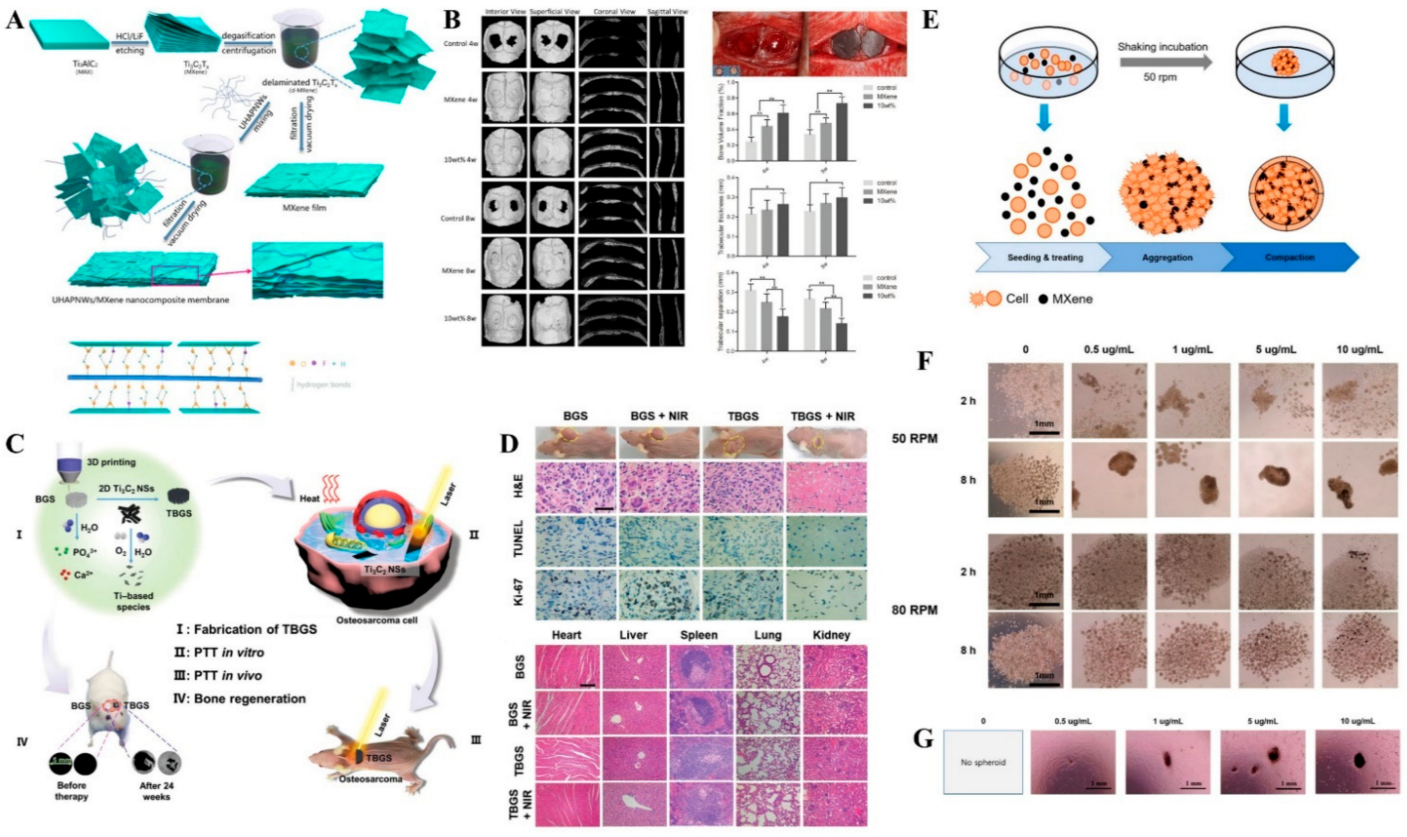
(**A**) Schematic illustration of the fabrication of hydroxyapatite nanowire/MXene (UHAPNWs/MXene) composite membrane displaying hydrogen bonding interaction within the composite. (**B**) Bone regeneration applications of UHAPNWs/MXene nanocomposite. Reprinted with permission from Ref. [198]. Copyright © 2022 Elsevier. (**C**) Schematic illustration of the fabrication of Ti_3_C_2_T_x_-bioactive glass scaffold for bone regeneration and ablation of cancer. (**D**) Tumor tissues stained by H&E, TUNEL, and Ki-67, and staining of heart, liver, spleen, lung, and kidney of tumor-bearing mice. Reprinted with permission from Ref. [189]. Copyright © 2019 Wiley. (**E**) Schematic illustration of the rapid production of cell spheroids using MXene. (**F**) Cell spheroid formation with varying MXene concentration and shaking speed. (**G**) Microscopic images of the cell migration and spheroid growth. Reprinted with permission from Ref. [185]. Copyright © 2021 MDPI.

Two-dimensional materials such as graphene and MoS_2_ are renowned for tissue engineering applications [179,180,181]. Recent years witnessed remarkable prospects for MXene in tissue engineering (Table 6) owing to its structural, biological, optical, electronic, and extraordinary physicochemical properties. Numerous investigations demonstrated that MXene scaffolds with stimulatory tissue regeneration effects, regulated discharge behavior, and NIR photothermal translation hold promise for tissue engineering [182] (Figure 11). Bone loss is regarded as a challenging aspect, where regenerating the alveolar bone imperfection is critical for the integration and functioning of the implant [182], and MXenes play an important role in guided bone regeneration through osteoinductivity [183]. MXenes such as Ti_3_C_2_T_x_ have been found to efficiently enhance the cell proliferation rate and osteogenic differentiation ability of the PLA scaffolds [184]. Recently, MXenes were found to enhance the production of cell spheroids. Lee et al. [185] reported enhanced production of mesenchymal stem cell spheroids using Ti_3_C_2_T_x_ MXene particles as a cell-adhesion agent (Figure 11E–G). The spheroid formation was induced within 6 h when a MXene concentration greater than 1 μg/mL was used, and the highest production rate was observed at the MXene concentration of 5 μg/mL. Furthermore, osteogenic differentiation was promoted by the produced spheroids without the requirement of an osteogenic medium. In one study, electrospun fibers of polycaprolactone-MXene induced biomineralization activity and resulted in hardened tissue formation [186]. Jin et al. [187] reported electrospun nanofibers of MXene/PLLA-PHA for cell culture and tissue engineering applications. The nanofibers reportedly accelerated the activity, proliferation, and osteogenic differentiation of BMSCs.

**Table 6:** Literature reports on the tissue engineering applications of MXene/Composites.

MXene/Composite	Applications	Description	Ref.
Ti_3_C_2_T_x_-enhanced poly (lactic acid) nanocomposite	Guided bone regeneration	Ti_3_C_2_T_x_-Poly (lactic acid) composite addition to MC3T3-E1 mouse preosteoblasts enhanced the in vitro adhesion, proliferation, and osteogenic differentiation.	[184]
Electrospun MXene/PLLA-PHA nanofibers	Cell culture	MXene composite nanofibers enhanced the differentiation of BMSCs to osteoblasts.	[187]
Ti_3_C_2_T_x_-PEG composite	Cardiac tissue engineering	3D-printed Ti_3_C_2_T_x_-PEG hydrogel aligned the iCMs with an increase in *MYH7*, *TNNT2*, and *SERCA2* expressions.	[188]
Ti_3_C_2_T_x_-Bioactive glass scaffold	Tissue reconstruction	MXene-bioactive glass scaffold demonstrated accelerated in vivo growth of newborn bone tissue.	[189]
Multilayered Ti_3_C_2_T_x_	Guided bone regeneration	Evaluated the guided bone regeneration ability of multilayered Ti_3_C_2_T_x_ in vitro and in vivo.	[190]
Ti_3_C_2_T_x_ Quantum Dots-Chitosan hydrogel	Tissue repair	MXene Quantum dot-chitosan hydrogel enhanced the physicochemical properties for tissue repair and stem cell delivery.	[191]
Mesoporous Silica@ Nb_2_C-Scaffolds	Nitric oxide-Augmented bone regeneration	NIR-triggered hyperthermia on the Nb_2_C MXene wrapped with S-Nitroso thiol-mesoporous silica with 3D-printing bioactive glass scaffolds and precisely released controlled nitric oxide.	[192]
Reduced graphene oxide-Ti_3_C_2_T_x_ hydrogel	3D cellular network formation	rGO-MXene hydrogel enhanced the formation of a 3D cellular network of human cell lines HeLa, SH-SY5Y, and MSU 1.1.	[193]
MXene-Hydroxyapatite nanoparticle composite	Osteogenic properties	MXene-Hydroxyapatite nanocomposite promoted the growth and osteogenic differentiation of BMSCs.	[194]
Ti_3_C_2_T_x_-CSH scaffold	Maxillofacial tissue regeneration	MXene-CSH scaffold stimulated the in vivo formation of maxillofacial bone, and induced the osteogenic protein expression of MC3T3-E1 in vitro.	[195]
Nb_2_C@Titanium plate	Tissue regeneration	The scavenging of excessive ROS from the infectious tissue environment by the Nb_2_C@Titanium plate alleviated the proinflammatory responses, thereby benefiting angiogenesis and tissue regeneration.	[196]
Ultrathin Ti_3_C_2_T_x_ nanoflakes	Periodontal regeneration	Human PDLCs pretreated with Ti_3_C_2_T_x_ displayed excellent in vivo new bone formation and enhanced osteoclast inhibition.	[197]

## 7. Summary and Outlook

Research on MXenes has seen a recent leap with an exponential increase in the number of research publications each year. Among the plethora of applications reported, biomedical applications have emerged as a promising avenue for these materials. A wide range of applications such as anticancer, drug delivery, antimicrobial, biosensing, and tissue engineering have been reported for MXenes. MXenes can be incorporated into textile fibers due to their transparency, thinness, conductivity, and mechanical strength for wearable diagnostics and fitness monitoring. Another important application aspect is electronic tattoos for monitoring physiological states. MXene-based sensor platforms are already being explored for quantifying attomolar concentrations of analytes, suggesting a bright future for next-generation biomarker determination. However, with the numerous possible MXene compositions and the unlimited number of solid solutions that can offer unique combinations of tunable properties by varying the ratios of ‘M’ and/or ‘X’ elements, only a handful of MXenes are widely explored. This discrepancy is particularly visible with the Ti_3_C_2_T_x_ Mxene, which accounts for more than 70% of all MXene-related research, indicating that MXene research is still in an early stage, and systematic guidelines are required for the use of MXene in biomedical applications. Additionally, the unique combinations of properties for this large, underexplored family of MXenes may open the door to a wide variety of biomedical applications. Even though in vitro cytotoxicity effects on delaminated Ti_3_C_2_T_x_ MXene are known, one particularly worrying scenario is that the long-term effects of MXenes on the human body are not fully understood since the physiological effects of MXenes and their composites are not fully explored, thereby making the long-term effects of many MXenes a mystery. The unexplored MXenes and their composites may accumulate in the body and may lead to potential toxicity. Since cytotoxicity of MXenes is influenced by the synthesis method, surface functionalities, and morphology, a universal claim of biocompatibility would be inappropriate. This is particularly important as more and more MXenes are making their way into POC devices and in vivo applications. Since this pathway is important to be fully understood for the materials to be deemed safe for clinical use, an in-depth mechanistic study of the cell growth in the presence of MXene may enable a realistic prediction of its cytotoxicity. The biocompatibility of MXenes may be further improved by coating them with a suitable polymer. The composite thus produced will alleviate the limitations of individual components with synergetic properties.

Simulation and modeling studies can offer insights into potential future applications of MXenes as well. Simulation studies such as Monte Carlo and molecular dynamics methods are already reported for investigating the adsorption of biomolecules on different functionalized MXenes. Similar strategies may be employed for exploring the interaction of MXenes and biomolecules to gain insights into the cytotoxicity effects and long-term stability of MXenes under in vivo conditions. Simulation studies may also become useful tools for addressing technological challenges such as storage technology for the efficient storage and improvement of oxidative and thermal stability, which is essential for enabling the full potential of MXenes for biomedical applications. Another challenging aspect is the synthesis part. Even though friendly approaches other than the fluoride etching are explored and reported, more environment-friendly preparation approaches need to be investigated to ensure minimal hazard to the environment upon release. Before large-scale and widespread manufacturing of MXene devices for health-related applications can be realized, improvements in synthesis, manufacturing, processing, and integration are necessary. A central issue with any MXene-based biosensor, particularly in competition with the existing technologies, is a low-cost, yet controllable, material synthesis route. The existing MXene synthesis methods that can provide minimal sample-to-sample variation are not compatible with polymeric flexible substrates due to the high-temperature requirement; therefore, growth is usually performed on a separate substrate, and then the 2D layer is transferred. Thus, an important research goal is to either improve/demonstrate the scalability and low-cost uniformity of transfer techniques or enable the synthesis directly onto the final substrate. Biofouling is another important concern since, in cases of in vivo applications, MXenes may encounter a complex biological matrix, which in turn may deteriorate the performance of the device in time. This, in turn, has been addressed to some degree by chemical modifications or coating with a polymer. While significant progress has been made in MXenes for biosensing and biomedical applications, commercial products in this arena are still largely pushed by small-scale startup companies, and further development for next-generation devices and applications may require a collaborative effort between academia and large industrial partners.

## Figures and Tables

**Table 1:** Literature reports of MXenes for drug delivery applications.

MXene-Based Drug Carrier	Stimuli for Drug Release	Drug	Advantages	Ref.
Ti_3_C_2_T_x_-SP	pH, NIR	Doxorubicin	High drug-loading capability of 211.8%.	[47]
Ti_3_C_2_T_x_-CoNWs	pH, NIR	Doxorubicin	High drug-loading capacity of 225.05%.	[49]
Ti_3_C_2_T_x_@GNRs/PDA/Ti_3_C_2_T_x_	NIR	Doxorubicin	95.88% drug-loading ability.	[50]
Ti_3_C_2_T_x_/Polyacrylamide	pH	Chloramphenicol	Ti_3_C_2_T_x_/Polyacrylamide hydrogels exhibited a high drug-loading of 97.5–127.7 mg/g and drug release percentages of 62.1–81.4%.	[53]
HAP/CS/HA/MXene/AuNRs	pH, NIR	Doxorubicin	Drug encapsulation efficiency of 83.9%	[54]
Polymer-coated MXene nanobelt fibers	NIR	Vitamin E	NIR-induced relaxation of the interface by the polymeric coating layer to dissolve and release Vitamin E.	[56]
Ti_3_C_2_T_x_@Agarose hydrogel	NIR	Doxorubicin	The DOX-loaded MXene-hydrogel exhibited rapid DOX release under NIR the irradiation, while almost no DOX release when NIR was turned off, proving an NIR switch for controlled drug release.	[57]
MXene@Agarose	NIR	HGF	Flexible and controllable release of the protein drugs with high precision.	[58]
MXenes-FA-SP	pH	Doxorubicin	Drug-loading capacity of 69.9% and 48 h long drug release time.	[59]
Ti_3_C_2_T_x_@Met@CP	pH, NIR	Metformin	The functionalized Ti_3_C_2_T_x_ nanosheets in the composite exhibited effective singlet oxygen generation, strong NIR absorption, and high photothermal conversion efficiency of ~59.6%.	[60]
Ti_2_N@oSi	NIR	Doxorubicin	Ultrahigh drug-loading capacity of 796.3%.	[61]
MXene@MOF-5@DOX	pH	Doxorubicin/pCRISPR	Achieved a drug payload of 35.7%.	[62]

Note: The Ti_3_C_2_Tx MXene, also referred to as Ti_3_C_2_ or Ti_3_C_2_Tz is unified by the term Ti_3_C_2_Tx in the main text to avoid confusion.

**Table 2:** Literature reports on the anticancer applications of MXenes and composites.

MXene/Composite	Anticancer Strategy	Advantages	Ref.
Nb_2_C nanosheets	Photothermal therapy	The surface-engineered Nb_2_C nanosheets feature low phototoxicity, high biocompatibility, biodegradability, and efficient in vivo photothermal ablation.	[45]
V_2_C Quantum dots	Photothermal therapy	V_2_C QDs photothermal agent combined with the low-temperature nucleus-targeted photothermal therapy mediated by engineered exosome vector for effective tumor eradication.	[73]
CGDSTC NSs	Photodynamic therapy	Sodium ascorbate and dopamine-modified Ti_3_C_2_T_x_ nanosheets conjugated with glucose oxidase and chlorin e6 photosensitizer for the efficient killing of cancer cells through cooperative effect.	[74]
Ti_3_C_2_T_x_-PVP@DOXjade	Chemo-photothermal therapy	The photoactivated DOXjade at the Ti_3_C_2_T_x_-PVP results in iron chelation and chemotherapeutic functions at the tumor sites. The MXene platform achieved a photothermal conversion efficiency of 40%.	[76]
Delaminated Ti_3_CN	Photothermal therapy	The photonic hyperthermia resulted in highly efficient tumor-killing both in vitro and in vivo.	[78]
Fe-Ti_3_C_2_T_x_	Chemodynamic, MIR, and photothermal therapy	Effective against MKN45 tumor in Balb/c nude mice.	[79]
Ti_3_C_2_T_x_-GOX-CPO/TPZ	Macrophage-mediated phagocytosis	A combination of enzyme dynamic therapy, tumor phototherapy, and hypoxia-activated chemotherapy for efficient tumor eradication.	[80]
2D ultra-thin Ti_3_C_2_T_x_	Photothermal therapy	Efficient photothermal therapy against MDA-231 breast cancer cells.	[81]
2D Nb_2_C MXenes	Chemo-photothermal therapy	A “therapeutic mesopore” layer is constructed on the surface of 2D Nb_2_C MXene, thereby supplementing the photothermal therapy with chemotherapy for enhanced ablation of U87 cancer cell line.	[82]
Mo_2_C nanospheres	Photodynamic-photothermal therapy	Biocompatible and multifunctional theranostic platform with minimal tissue toxicity for effective in vivo tumor depiction.	[83]

**Table 3:** Literature reports on the antimicrobial applications of MXene/Composites.

MXene/Composite	Antimicrobial Applications	Ref.
Ti_3_C_2_T_x_	Antibacterial activity against *E. coli* and *B. subtilis* with 98% viability loss within 4 h.	[84]
Colloidal Ti_3_C_2_T_x_	Antibacterial activity against *B. subtilis* and *E. coli.*	[85]
Ti_3_C_2_T_x_	Antibacterial activity against *E. coli.*	[87]
Ti_3_C_2_T_x_	Photocatalytic inactivation of airborne *E. coli.*	[93]
Bi_2_S_3_/Ti_3_C_2_T_x_	Photoexcited antimicrobial effects on *S. aureus* and *E. coli.*	[94]
Ti_3_C_2_T_z_/Chitosan	Antibacterial activity against *E. coli* and *S. aureus.*	[95]
Nb_2_CT_x_ and Nb_4_C_3_T_x_	Bactericidal property against *E. coli* and *S. aureus.*	[96]
Cu_2_O/Ti_3_C_2_T_x_	Antibacterial activity against *S. aureus* and *Pseudomonas aeruginosa.*	[97]
Ti_3_C_2_T_x_-AuNCs	Antibacterial performance on *S. aureus* and *E. coli.*	[98]
MoS_2_/Ti_3_C_2_T_x_	Antibacterial activity against *E. coli* and *B. subtilis.*	[99]
Ti_3_C_2_T_x_-Laden bacteriophage	Antibacterial activity against *Shigella.*	[100]
Ag/Ti_3_C_2_T_x_	Inhibitory activity against *E. coli* and *S. aureus.*	[101]
TiVCT_X_	Antibacterial activities against *E. coli*, photothermal sterilization effect on *E. coli* and *B. subtilis.*	[102]
CuP-sTi_3_C_2_T_x_	Antibacterial activity against *E. coli* and *S. aureus.*	[103]
Ti_3_C_2_T_x_	Size-dependent photothermal antibacterial activity against *S. aureus.*	[104]
Ti_3_C_2_T_x_/PVA hydrogel	Antibacterial activity against *E. coli* and *S. aureus.*	[105]
V_2_C NSs	Antibacterial activity against *E. coli*, and *B. subtilis.*	[106]
BC/Chi/Ti_3_C_2_T_x_/AgNWs aerogel	Antibacterial activity against *E. coli* and *S. aureus.*	[107]

**Table 4:** Literature reports on the MXene-based smart devices.

Sensing Platform	Device Type	Applications	Ref.
PDA-MXene-PDMS	Wrist band	Body motion monitoring	[125]
MXene/Prussian blue	Wrist band	Perspiration analysis	[130]
MXene/MWCNT electronic fabric	Mask	Respiration analysis	[131]
Ti_3_C_2_T_x_/PVDF-TrFE	Patch	Acquisition of physiological signal	[133]
MXene/CNC coated TPU NWF	Patch	Strain/Pressure sensor	[134]
MXene coated NWF	Fabric	EMI Shielding, Wearable heater	[135]
MXene coated cotton	Fabric	Pressure sensor	[136]
rGO/Ti_3_C_2_T_x_	Fabric	Human motion monitoring	[137]
SnS/Ti_3_C_2_T_x_ nanohybrid	Patch	Sitting posture analysis and sign-to-text translation	[138]
MXene-based core-sheath yarns	Knitted band	Strain/Humidity sensor	[139]
MXene coated cellulose	Knitted wrist band	Pressure sensor	[140]
Ag/MXene nanocomposite	Patch	Strain Sensor	[141]
MXene-NSD-PEDOT	Patch	EMG signal analysis	[142]
MXene/PU composite fibers	Knitted band	Strain sensor	[143]

**Table 5:** Literature reports on MXene-based biomarker detection platforms.

MXene-Based Sensor Platform	Sensing Technique	Biomarker	Linear Range	LOD	Ref.
Ti_3_C_2_T_x_@CuNCs/MB	Photothermal	Human anti-ASGPR	10^−8^ U/mL	1.76 × 10^−9^ U/mL	[150]
			-		
			10^−2^ U/mL		
BSA/anti-CEA/f-Ti_3_C_2_T_x_-MXene/GC	CV	CEA	0.0001	0.000018 ng/mL	[151]
			-		
			2000 ng/mL		
Ag@Ti_3_C_2_T_x_	Fluorometric	NSE	0.0001	0.05 pg/mL	[152]
			-		
			1500 ng/mL		
Chi/ChOx/Ti_3_C_2_T_x_	DPV	Cholesterol	0.3–4.5 nM	0.11 nM	[153]
Ti_3_C_2_T_x_-AFBPB-film modified DIDμE	DPV	Apo A1	0.1 pg/mL	0.3 pg/mL	[154]
			-		
			50 ng/mL		
		NMP22	0.1 pg/mL	0.7 pg/mL	
			-		
			50 ng/mL		
N-Ti_3_C_2_T_x_-MXene	SPR	CEA	10^−11^–10^−6^ g/mL	1.7 pg/mL	[155]
DNA modified Ti_3_C_2_T_x_ nanosheets	FRET	miRNA-21 a	5 fM–100 pM	0.62 fM	[156]
		miRNA-10 b	5 fM–100 pM	0.85 fM	
PPy@Ti_3_C_2_T_x_/PMo_12_	EIS	OPN	0.05–10,000 pg/mL	0.98 fg/mL	[157]
Ti_3_C_2_T_x_/AuNPs/SPA/Ab_1_	SPR	CEA	2 × 10^−16^	0.07 fM	[158]
			-		
			2 × 10^−8^ M		
MoS_2_@Ti_3_C_2_T_x_	DPV	Thyroxine	0.78	0.39 pg/mL	[159]
			-		
			7.8 × 10^6^ pg/mL		
ssDNAs/AuNP@Ti_3_C_2_T_x_/SPGE	DPV	miRNA-21	500 aM	204 aM	[160]
			-		
			50 nM		
		miRNA-141	500 aM	138 aM	
			-		
			50 nM		
cDNA-Fc/Ti_3_C_2_T_x_/Apt/Au/GCE	DPV	MUC1	1 pM–10 µM	0.33 pM	[161]
cDNA/Ti_3_C_2_T_x_@FePcQDs/AE	EIS	miRNA-155	0.01 fM	4.3 aM	[162]
			-		
			10 pM		
MB/DNA/HT/HP1/AuNPs/Ti_3_C_2_T_x_/BiVO_4_/GCE	PEC	VEGF_165_	10 fM	3.3 fM	[163]
			-		
			100 nM		
GSH-Ti_3_C_2_T_x_ MQDs	FRET	UA	-	125 nM	[164]
SOx/Ti_3_C_2_T_x_-Chi/GCE	CA	Sarcosine	36–7800 nM	18 nM	[165]
Hb-Ti_3_C_2_T_x_-GO/Au	DPV	H_2_O_2_	2 μM–1 mM	1.95 μM	[166]
Ti_3_C_2_T_x_/GCPE	CA	Adrenaline	0.02–10 μM, 10–100 μM	9.5 nM	[167]
Ti_3_C_2_T_x_-HF/TBA	CA	Glucose	50–250 μM	23 μM	[168]
Urease-MB/Ti_3_C_2_T_x_/SPE	SWV	UA	30–500 μM	5 μM	[169]
		Urea	0.1–3 μM	0.2 μM	
L-cys/AuNPs/Ti_3_C_2_T_x_	CA	Sweat cortisol	5–40 ng/mL	0.54 ng/mL	[170]
			40–180 ng/mL		
Au-Pd-Pt/Ti_3_C_2_T_x_	DPV	CEA	1 fg/mL	0.32 fg/mL	[171]
			-		
			1 ng/mL		

## Data Availability

Not applicable.

## Abbreviations

Ab1	Monoclonal anti-CEA antibody
AChE	Acetylcholinesterase
AFBPB	4-amino-1-(4-formyl-benzyl) pyridinium bromide
AgNPs	Silver nanoparticles
AgNRs	Silver nanorods
AMF	Alternating magnetic field
anti-ASGPR	Anti-asialoglycoprotein receptor
anti-CEA	Anti-Carcinoembryonic antigen
Apo A1	Apolipoprotein A1
Apt	Aptamer
ATCl	Acetylthiocholine chloride
AuNPs	Gold nanoparticles
Au-PCB	Gold printed circuit board
BC	Bacterial cellulose
BMSCs	Bone marrow derived mesenchymal stem cells
BSA	Bovine serum albumin
CA	Chronoamperometry
cDNA	Complementary DNA
CGDSTC NSs	Chlorin e6/GOx/Dopamine/Sodium ascorbate/Ti_3_C_2_T_x_ nanosheets
Chi	Chitosan
ChOx	Cholesterol Oxidase
CNC	Cellulose nanocrystal
CNTs	Carbon nanotubes
CP	Compound polysaccharide
CPO	Chloroperoxidase
Cre	Creatinine
CSH	Collagen/Silk/Hydroxyapatite
CuP	Copper-organophosphate
CV	Cyclic Voltammetry
CVD	Chemical Vapor Deposition
DA	Dopamine
DIDμE	Dual interdigitated microelectrode
DMM	Digital multimeter
DNA	Deoxyribonucleic acid
DPV	Differential Pulse Voltammetry
EIS	Electrochemical Impedance Spectroscopy
EMG	Electromyography
FA	Folic acid
Fc	Ferrocene
FePcQDs	Phthalocyanine quantum dots
FRET	Fluorescence Resonance Energy Transfer
GA	Glutaraldehyde
GCE	Glassy carbon electrode
GCPE	Graphite Carbon Paste Electrode
GDH	Glutamate dehydrogenase
GO	Graphene oxide
GONR	Graphene oxide nanoribbon
GSH	Glutathione
H&E	Hematoxylin and eosin
H_2_O_2_	Hydrogen peroxide
Hb	Hemoglobin
HF	Hydrofluoric acid
HGF	Hepatic growth factor
HP1	Hairpin DNA
HRP	Horseradish peroxidase
HT	Hexane thiol
IC	Ion chromatography
LOD	Limit of detection
MB	Methylene blue
miRNA-21	micro-Ribonucleic acid-21
miRNA-10b	micro-Ribonucleic acid-10b
miRNA-141	micro-Ribonucleic acid-141
miRNA-155	micro-Ribonucleic acid-155
MQDs	MXene quantum dots
MUC1	Mucin 1
MWCNT	Multi-walled carbon nanotubes
MXNSs	2D MXene-Ti_3_C_2_T_x_ nanosheets
NAD	Nicotinamide adenine dinucleotide
NIR	Near Infrared
NMP22	Nuclear Matrix Protein22
NSE	Neuron specific enolase
NWF	Non-woven fabric
OPN	Osteo5pontin
P(DPA)	Poly (di picolinic acid)
PAYR	Poly alizarine yellow R
PB	Prussian blue
Pd	Palladium
PDA	Polydopamine
PDLCs	Periodontal ligament cells
PDMS	Polydimethylsiloxane
PEC	Photoelectrochemical
PEDOT	Poly(3,4-ethylenedioxythiophene)
PEG	Polyethylene glycol
PEPLD	Plasma-enhanced pulsed laser deposition
PHA	Poly hydroxy alkanoate
PLLA	Poly (L-lactic acid)
PMo_12_	Phosphomolybdic acid
POC	Point-of-care
PPy	Polypyrrole
PSA	Prostate specific antigen
PtNPs	Platinum nanoparticles
PU	Polyurethane
PVA	Polyvinyl alcohol
PVDF	Polyvinylidene fluoride
QDs	Quantum dots
rGO	Reduced graphene oxide
ROS	Reactive oxygen species
Ru	Ruthenium
SED	Soft electronic devices
SEM	Scanning electron microscopy
SOx	Sarcosine oxidase
SPA	Staphylococcal protein A
SPE	Screen printed electrode
SPEEK	Sulfonated PEEK substrates
SPGE	Screen-printed gold electrode
SPR	Surface plasmon resonance
ssDNAs	Single stranded DNAs
SWV	Square wave voltammetry
TBA	Tetrabutylammonium
TGA	Thio glycolic acid
TPU	Thermoplastic polyurethane
TPZ	Tirapazamine
TrFE	Trifluoro ethylene
TUNEL	Terminal deoxynucleotidyl transferase dUTP nick end labeling
Tyr	Tyrosine
UA	Uric acid
UHAPNWs	Ultralong hydroxyapatite nanowires
VEGF165	Vascular endothelial growth factor 165
β-HBD	β-hydroxybutyrate dehydrogenase
